# Association of Blood Heavy Metal Levels and Renal Function in Korean Adults

**DOI:** 10.3390/ijerph19116646

**Published:** 2022-05-29

**Authors:** Yoonjin Park, Su-Jung Lee

**Affiliations:** 1 Department of Nursing, Joongbu University, Geumsan-gun 32713, Korea; pyj2272@naver.com; 2 School of Nursing, Research Institute of Nursing Science, Hallym University, Chuncheon-si 24252, Korea

**Keywords:** lead, mercury, cadmium, nickel, renal function

## Abstract

This study aimed to investigate the association between the levels of lead, mercury, cadmium, and nickel in the blood and renal function and to provide basic data for the development of health programs for the prevention of renal failure. This study included 1984 participants aged 19 and older who participated in the Korean National Health and Nutrition Examination Survey from 2019. Differences in values according to general characteristics and the estimated glomerular filtration rate (eGFR) stage were analyzed using a chi-square test, t-test, ANOVA, and the influencing factors were analyzed through multiple regression analysis. The concentrations of lead, mercury, cadmium, and nickel, and the risk of elevated eGFR were analyzed using linear regression. The correlation between the variables was determined using Pearson’s correlation analysis. Analysis of the correlation between blood lead, mercury, cadmium, and nickel levels and blood eGFR levels revealed that blood eGFR levels were significantly negatively correlated with blood lead, cadmium, and nickel levels (*p* ≤ 0.001). This study is significant in that it found a significant association between decreased eGFR and heavy metal levels and provided meaningful basic data on the association between heavy metals and renal function.

## 1. Introduction

With advanced industrialization, various heavy metals are increasingly threatening human health through an array of routes such as food, industrial products, soil, air, and water quality. In particular, environmental pollution shows that diversification, accumulation, and ecological destruction negatively affect human health [1]. Since 2009, South Korea has conducted a survey every three years for basic data to establish environmental health policies, such as the in vivo concentration of environmentally harmful factors, status of health hazards caused by environmentally harmful factors, and occurrence of environmental diseases. Since 2021, the number of hazardous substances has greatly expanded from the existing 33 types to 64 types, the concentration of hazardous substances has been determined, and the causes of exposure to hazardous substances have been identified [2]. When heavy metals enter the body, some accumulate without being excreted, and even a small amount has a harmful effect on health [3].

In addition to occupational exposure, heavy metals can chronically accumulate in the body through food, water, air, and the skin and may affect the blood, lungs, kidneys, and liver [4]. In particular, the kidneys can become target organs for heavy metal toxicity due to their ability to reabsorb and concentrate ions and metals [5]. Moreover, the kidney is a major excretory organ for lead, and continuous exposure to lead is likely to cause renal proximal tubular changes. Kidney damage depends on the amount, duration, and nature of heavy metal exposure [6]. Cadmium is commonly used in pigments, batteries, and alloys of other metals. Phosphate fertilizers are one of the causes of cadmium exposure, and the ingestion of crops grown in contaminated soil can increase cadmium levels in the blood [7]. Immediately following its absorption in the body, cadmium combines with metallothionein and is stored in red blood cells, which increases renal toxicity. The half-life in the body is 10 to 30 years, and low concentrations of exposure tend to accumulate in the renal cortex, reducing the glomerular filtration rate and resulting in proteinuria [8]. Chronic exposure to mercury may lead to its accumulation in the kidneys and cause epithelial damage and necrosis in the proximal tubules and rectum [5].

Furthermore, nickel is used to make corrosion-resistant alloys such as stainless steel, which is commonly found in daily life; long-term exposure to nickel may cause a decline in renal function [9]. The Korea Centers for Disease Control and Prevention (KCDC) also recognize that continuous exposure to heavy metals may negatively affect health. The KCDC continues to conduct research on related diseases by including collected data on lead, mercury, and cadmium in the fourth Korean National Health and Nutrition Examination Survey (KNHANES) since 2005 and has collected data on nickel in the KNHANES since 2016 [10]. In South Korea, the proportion of patients on dialysis has doubled over the past decade. In 2019, the total number of patients with end-stage renal failure exceeded 100,000, of which 81,760 (75.1%) received hemodialysis treatment [11]. The decrease in renal function progresses slowly for several years, and the symptoms are generally mild, and in most cases is recognized when renal function decline has progressed significantly; thus, it may be difficult to recover renal function [12]. However, early detection and intervention can prevent chronic kidney disease, and when management strategies are implemented, the incidence of end-stage kidney disease is reduced [13]. Recent studies on chronic kidney disease reported that environmental exposure to heavy metals is a potential risk factor for chronic kidney disease [14]. Therefore, studies on the association between heavy metals and renal function are very important in modern society and are essential for long-term health management. The aim of the study was to investigate the association between the levels of lead, mercury, cadmium, and nickel in the blood and renal function by calculating eGFR level. In addition, this study provides basic data for the development of health programs for the prevention of renal failure by analyzing factor that affect eGFR level.

## 2. Materials and Methods

### 2.1. Study Design

The National Health and Nutrition Survey was conducted every three years from 1998 to 2005 and has been conducted annually since 2007 to improve national statistics timelines. The purpose of the KNHNES is to calculate statistics with national representation and reliability regarding people’s health, health behavior, and food and nutrition intake, and to use them as basic data for health policies such as setting and evaluating goals for the National Health Promotion Plan. The heavy metal test of this survey investigated 2000 people aged 20 years or older in 2008–2009, 2400 people aged 10 years or older in 2010–2013, and 1/3 of those aged 10 years or older after 2016. Moreover, since this survey was conducted through a sampling method of stratifying the entire population of the Republic of Korea and sampling colonies, the KNHANES data used for the analysis were extracted using multi-stage clustered probability sampling, which is a complex sample design intended to improve the representativeness of the sample and estimation accuracy for the primary sampling units (PSUs). The types of weights in the KNHNES can be largely classified into household weights for household unit analysis and individual weights for individual unit analysis. Household weight was assigned to households participating in the survey to represent all households in South Korea, and individual weight was assigned to individuals participating in the survey to represent the entire population of South Korea. Furthermore, the individual weight was controlled by forming a separate weight classified by survey sector owing to the difference in the number of participants by survey sector. This study was exempted from IRB review (IRB number: JIRB-2021110801-01) and raw data were downloaded from the Korea Disease Control and Prevention Agency KNAHES website (https://knhanes.kdca.go.kr/knhanes/main.do (accessed on 2 January 2022)).

### 2.2. Sample

**Figure 1:**
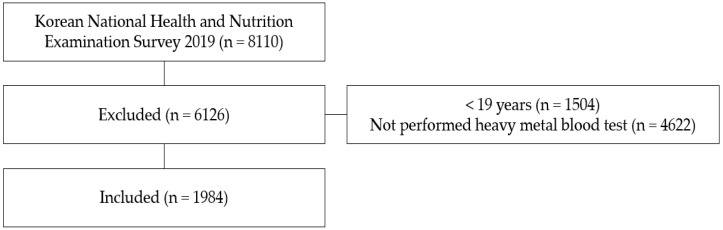
Simplified flow chart of study subject selection.

A total of 192 PSUs were selected annually, from which facilities such as military facilities, prisons, and foreign households were excluded; 25 final target households were selected for each PSU using systematic sampling since the first year of the eight KNHANES (2019). For the appropriateness of the sample, differences in the number of households and populations, regional differences, and non-response errors of non-participants were corrected to increase the representation and accuracy of the target population, 1South Koreans’ health behavior, chronic disease prevalence, and food and nutrition. The target participants in the first year of the eighth KNHANES were 10,859 individuals, of whom 8110 participated in at least one health interview, health examination, and nutrition survey, with a participation rate of 74.7%. Heavy metal testing was conducted for some participants selected from among those who underwent health examinations, and a weight was used for calculation. This study analyzed 1984 individuals aged 19 years or older who underwent heavy metal testing (Figure 1).

### 2.3. Measures

#### 2.3.1. Demographic and Disease Characteristics

The general participant characteristics included gender, income level, and education level. Income level was divided into low and middle low, middle high and high. Education level was divided into ≤9 and ≥10 years. Moreover, Smoking was defined as having smoked more than 100 cigarettes in one’s lifetime or being a current smoker. Comorbidities were categorized into hypertension, diabetes mellitus. Among the examination items, this study used height, weight and blood pressure, which were measured by a trained examiner. Anthropometry included anthropometry (height, weight), blood pressure (systolic and diastolic).

#### 2.3.2. Biochemical Measurements

Blood samples were collected mainly via the median cubital vein or cephalic vein in each participant after fasting for at least eight hours. The blood samples were refrigerated and delivered to the clinical laboratory on the same day for analysis within 24 h. Triglycerides, high-density lipoprotein cholesterol (HDL-C), and hemoglobin A_1_C (HbA_1_C) levels were measured by enzymatic methods using a Hitachi automatic analyzer 7600 (Tokyo, Japan). Heavy metals were analyzed by atomic absorption spectrophotometry using a PerkinElmer AAFA600 instrument.

#### 2.3.3. Renal Function

The Modification of Diet in Renal Disease (MDRD) formula equation based on information on creatinine levels, age, sex, and race was used [15] estimated glomerular filtration rate (eGFR) (ml/min/1.73 m^2^) = 186 × (Scr) − 1.154 × (age) − 0.203 × 0.742 [if female]). The Korean Society of Nephrology classified renal function as follows [16]: stage 1 (eGFR ≥ 90, mL/min/1.73 m^2^), stage 2 (eGFR 60–90/mL/min), stage 3 (eGFR 30–59/mL/min), stage 4 (eGFR 15–29/mL/min), and stage 5 (eGFR < 15 mL/min). In this study, renal function was divided into stages 1, 2 and 3–5 according to the classification criteria of the Korean Society of Nephrology; stage 3 or higher was defined as the decreased renal function group for analysis.

### 2.4. Analytic Strategy

Data analysis was performed using SPSS version 22 (IBM Corp., Armonk, NY, USA). The statistical significance level was set at *p* value < 0.05. The KNHANES data used for the analysis were extracted using multi-stage clustered probability sampling, which is a complex sample design intended to improve the representativeness of the sample and estimation accuracy for the PSUs. Data analysis was performed with weights using the SPSS complex sample analysis method. The weight in the KNHANES was a raising multiplier, which was assigned to represent the entire Korean population, and was calculated by reflecting the extraction rate, response rate, and population distribution. Moreover, when new variables were created by combining several variables, or when a statistical model that used several variables for simultaneous analysis was fitted, appropriate weights were selected and applied, considering related categories, areas, and items for all analyzed variables. Differences in values according to general characteristics and eGFR stage were analyzed using the chi-square test, t-test, and ANOVA. The concentrations of lead, mercury, cadmium, and nickel and the risk of elevated eGFR were analyzed using linear regression. Correlations between variables were determined using Pearson’s correlation analysis. In addition, we performed multivariate regression analysis to identify factors affecting eGFR level. As a result of the univariate regression analysis, independent variables with a significance level of less than 0.05 were included in the multivariate regression analysis, and the statistical significance level was set to less than 0.05.

## 3. Results

### 3.1. Demographic and Clinical Characteristics

A total of 1984 people participated in this study, consisting of 920 men and 1064 women. The mean ages of the men and women were 48.33 and 48.20 years old, respectively. In terms of education level, the proportion of male and female with a length of education ≥ 10 years was the highest at 70.71% and 76.82%, respectively. In terms of Body Mass Index and hypertension were significantly different between male and female (*p* < 0.001 and *p* = 0.002), but there was no significant difference in diabetes by gender (*p* = 0.091). Systolic and diastolic blood pressure also showed significant differences according to gender (all *p* < 0.001). The mean HbA_1_C levels in male and female were 5.73 ± 0.911 and 5.60 ± 0.751, respectively, indicating a statistically significant difference (*p* < 0.001). Statistically significant differences exist between gender in triglyceride, HDL cholesterol (all *p* < 0.001). The average triglyceride level was 163.76 ± 37.66 for male and 109.13 ± 72.21 for female, which was higher in male. The mean HDL-C level was 46.91 ± 11.22 and 54.99 ± 12.44 in male and female. Gender comparison was also significant (all *p* < 0.05) in other blood test results (e.g., Lead level, Mercury, Cadmium, Nickel, and eGFR). Here, except for Cadmium, nickel and eGFR, mean scores were higher in female than in male (Table 1).

### 3.2. General Characteristics according to eGFR Stage

In terms of the mean age of the participants according to eGFR stage, the mean ages of those in the stage 1, 2 and 3–5 groups were 42.06, 52.9, and 67.39 years, respectively. When comparing eGFR by stage, the proportions of current smokers in the stage 1, 2 and 3–5 groups were 22.8%, 17.4%, 16.5%, respectively. The prevalence rates of hypertension in the stage 1, 2 and 3–5 groups were 11.0%, 22.7%, and 72.2%, respectively. The prevalence rates of diabetes in the stage 1, 2 and 3–5 groups were 6.1%, 9.0%, and 34.2%, respectively (*p* < 0.001). Thus, a lower eGFR was significantly associated with increased morbidity (*p* < 0.001). The mean HDL cholesterol levels in the stage 1, 2 and 3–5 groups were 51.97 mg/dL, 51.13 mg/dL, and 43.62 mg/dL mg/dL, respectively.

The mean blood lead levels by eGFR stage in the stage 1, 2 and 3–5 groups were 1.48 µg/L, 1.74 µg/L and 2.01 µg/L, respectively. The mean blood mercury levels in the stage 1, 2 and 3–5 groups were 3.56 µg/L, 3.89 µg/L, and 3.36 µg/L, respectively, indicating that there was statistically significant difference (*p* < 0.05). The mean blood cadmium levels in the stage 1, 2 and 3–5 groups were 0.90 µg/L, 1.01 µg/L, and 1.27 µg/L, respectively. The mean blood nickel levels in the stage 1, 2 and 3–5 groups were 0.32 µg/L, 0.32 µg/L, and 0.39 µg/L, respectively. Thus, a lower eGFR was significantly associated with higher blood levels (Table 2).

### 3.3. Factors Affecting Subjects’ eGFR

We conducted a univariate analysis of the variables that showed differences according to eGFR stage. And as a result of univariate analysis, multiple regression analysis was performed using the input method for variables with *p*-values less than 0.05. Multiple regression analysis was performed with gender, age, education status, household income, HbA1c, body mass index, systolic blood pressure, diastolic blood pressure, lead, mercury cadmium and nickel. Of these, the categorical variables, i.e., gender, education status, household income and current smoking, were treated as a dummy variable; the gender was female, education status was 10 years or more, household income was upper middle to highest and current smoking was non-smoker. When the Durbin-Watson correlation coefficient was checked to verify the basic assumptions of the regression analysis on eGFR, there was no autocorrelation with the coefficient 2.032. When the tolerance limit and the variance inflation factor (VIF) for the multicollinearity test were measured, the tolerance limit was 0.505~0.933, which was higher than 0.1, and the dispersion expansion factor was 1.071~2.111, which was less than 10, confirming that there was no problem in terms of multicollinearity. Through a multiple regression analysis, it was determined that the model was significant (F = 22.160, *p* = <0.001) and showed 26.6% of variance. Through multiple regression analysis, it was found that age (β = −0.492, *p* < 0.001), education level (β = −0.165, *p* < 0.001), diastolic blood pressure (β = −0.116, *p* < 0.001), blood nickel level (β = −0.107, *p* = 0.001), current smoking ((β = −0.086, *p* = 0.014), body mass index (β = −0.098, *p* = 0.004), and blood mercury level (β = 0.087, *p* = 0.009) had an effect on eGFR (Table 3).

### 3.4. Correlation between eGFR and Blood Lead, Mercury, Cadmium, and Nickel

**Figure 2:**
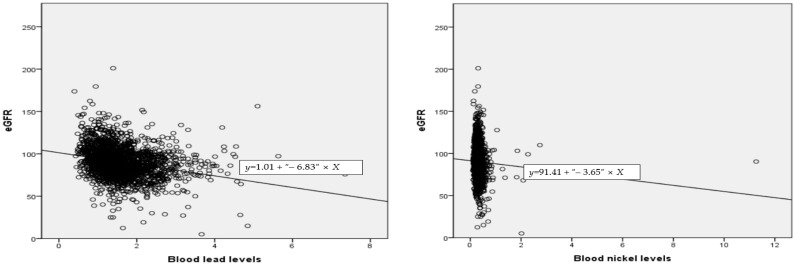

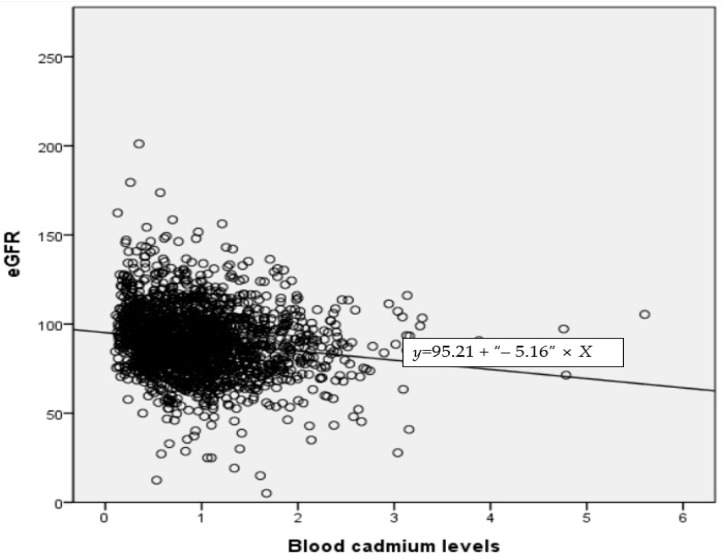
Scatter plot of blood lead, nickel, and cadmium levels and eGFR.

Analysis of the correlation between blood lead, mercury, cadmium, and nickel levels and blood eGFR levels revealed that blood eGFR levels were significantly negatively correlated with blood lead (r = −0.255, *p* ≤ 0.001), cadmium (r = −0.164, *p* ≤ 0.001), and nickel levels (r = −0.056, *p* ≤ 0.05). However, there was no significant correlation between eGFR and blood mercury levels (*p* > 0.05) (Table 4) (Figure 2).

### 3.5. Heavy Metal Factors Affecting Blood eGFR

Analysis of the factors affecting blood eGFR levels and blood lead, mercury, cadmium, and nickel levels using linear regression analysis revealed that the factors affecting eGFR in men were lead, cadmium, and nickel (F = 28.875, Adj. R^2^ = 0.095, *p* < 0.05); the autocorrelation coefficient of errors obtained by Durbin–Watson to test the assumption of the regression analysis was 1.973, indicating that there was no autocorrelation. The factors affecting eGFR in women were found to include blood lead and cadmium (F = 14.056, Adj. R^2^ = 0.054, *p* < 0.05); the autocorrelation coefficient of errors obtained with Durbin–Watson to test the assumption of the regression analysis was 2.061, indicating that there was no autocorrelation (Table 5).

## 4. Discussion

This descriptive study aimed to investigate the effects of heavy metals such as lead, mercury, cadmium, and nickel on renal function. Long et al. classified Chinese people into healthy, risk, and chronic kidney disease groups using eGFR values, and consequently confirmed the difference in eGFR according to gender; women had a higher eGFR than men in all three groups. However, in healthy and high-risk groups, the decline in eGFR was lower than that in women as age increased [17]. This gender difference can be thought of as the effect of hormones on glomerular structure, hemodynamic condition, and kidney cells [18]. It has been reported that men have a higher incidence and prevalence of terminal renal failure than women [19]. However, the progression rate of CKD showed gender differences according to age. Men showed a significant eGFR decline at the age of 20 to 40 years, while women showed a significant eGFR decline after menopause [20]. There, these gender differences are not limited to biological differences, but should be carefully interpreted considering various factors such as chronic glomerulonephritis, chronic epileptic nephritis, diabetes, age, and lifestyle [21,22].

According to previous studies similar to this study [23], the concentration of heavy metals in the blood differs according to gender, with men showing an especially high frequency. Therefore, this study investigated the relationship between heavy metals and kidney function. Uncontrolled blood pressure is an important risk factor for renal failure. It was reported that the progression to renal failure was more delayed in those whose blood pressure was controlled at 125/75 mmHg or lower (mean blood pressure: 92 mmHg) than in those whose blood pressure was controlled at 140/90 mmHg (mean blood pressure: 107 mmHg) [24]. The study also found that, in terms of eGFR divided into three stages, a higher stage was associated with elevated systolic blood pressure, and the systolic blood level was the highest in those with an eGFR ≤ 30. The seventh Joint National Committee report recommended that blood pressure in patients with chronic renal disease be controlled to ≤130/85 mmHg, and in particular, blood pressure in those with proteinuria ≥1.0 g per day be controlled to approximately 125/75 mmHg [25]. Therefore, controlling blood pressure is a key factor in the prevention of nephropathy [24].

As a result of multiple regression analysis of this study, the general variables affecting eGFR were gender, age, education level, household income, smoking status, body mass index, and diastolic blood pressure. This is similar to studies that affect age, body mass index, proteinuria, high blood pressure, anemia, etc. and diabetic kidney disease was affected by gender, education level, income level, drinking, smoking status, HbA_1_C, and blood sugar [26,27]. High blood pressure in chronic kidney disease negatively affects chronic kidney disease, and groups managed with appropriate blood pressure support an existing study that showed a 42% decrease in kidney disease compared to those who did not [28]. According to the results of this study, mercury and nickel were found to have an effect on eGFR in multivariate analysis. Chronic exposure to mercury can lead to accumulation of mercury in the kidneys, causing epithelial damage and necrosis in proximal tubules. Prolonged exposure to nickel can lead to deterioration of kidney function [5]. Therefore, the results of multiple regression analysis showed that mercury and nickel were very important variables to affect the kidney negatively.

Furthermore, this study found significant differences in the values and correlations between eGFR and lead, cadmium, and nickel levels (*p* < 0.05). Lead can be absorbed by the body in various forms, such as paints, cosmetics, and fuels. Consequently, lead may affect the gastrointestinal, hematopoietic, reproductive, and immune systems. The kidney, which is the route of lead excretion, is a target organ for lead toxicity, and can promote kidney damage by causing oxidative stress and lipid peroxidation in the kidney [29]. The results of the analysis revealed that lead was a significant influencing factor in both men and women. The mean blood lead levels were 1.85 ± 0.73 in men and 1.43 ± 0.61 in women. This is similar to 1.54 μg/dL in women and 1.82 μg/dL in men, as reported in a study regarding blood heavy metal concentrations among general adults conducted by the Ministry of Environment (2020) [30]. In addition, this study found that as blood lead levels increased, eGFR decreased. Lead levels had a significant positive correlation with blood mercury and cadmium levels, indicating that even a small amount of lead in the blood can be a very important indicator. A cohort study in the US reported that participants with a blood lead level ≥0.17 μmol/L had a cardiovascular disease mortality rate 1.55 times higher than those with a blood lead level of 0.09 μmol/L, and that blood lead levels were associated with both cardiovascular disease mortality and stroke mortality. Therefore, careful monitoring of other diseases and renal failure associated with blood lead levels is required [31]. According to the European Food Safety Authority (EFSA), exposure limits for adults are 1.2 to 4.2 (cardiovascular effects) and 0.51 to 1.8 (height toxicity); thus, low levels of lead cannot be overlooked [32].

This study also showed a significant correlation between blood cadmium levels and eGFR (*p* < 0.05). The primary organs accumulating cadmium are the liver and kidneys. Cadmium accumulated in the kidneys is reabsorbed in the proximal tubule, thereby increasing the amount of accumulated cadmium. The American Conference of Governmental Industrial Hygienists defined the biological exposure index as a blood cadmium level of 5 μg/L and a urinary cadmium level of 5 μg/g and paid early attention to renal function associated with chronic exposure to a very small amount of cadmium [33]. A previous domestic study also reported that the mean cadmium levels in the kidney cortex and liver among Koreans who had no occupational exposure to cadmium were 27.4 μg/g wet weight and 3.1 μg/g wet weight, respectively, and the cadmium levels in the kidney cortex were about nine times higher than in the liver; the study recommended vigilance to the risk of kidney disease associated with cadmium [34]. The results of this study also showed that there was a negative correlation between blood cadmium levels and eGFR, indicating that caution is required regarding the effect of cadmium exposure on renal function. Furthermore, a mixture of cadmium and lead appears to have a more pronounced effect on renal function than single exposure [29]. However, studies on the interaction between several other heavy metals are lacking [35], suggesting a need for future research on how the interaction between heavy metals affects other organs, including renal function. Since a previous study reported that chronic exposure to nickel leads to respiratory complications and renal dysfunction [36], this study also showed that there was a significant correlation between blood nickel levels and decreased eGFR (*p* < 0.05). Although there was no significant correlation between mercury and GFR in this study *(p* = 0.061), it cannot be overlooked, as an indirect but significant correlation can be seen with lead, mercury, and nickel, which negatively affects renal function.

A linear regression analysis was conducted to analyze the factors influencing blood eGFR and blood lead, mercury, cadmium, and nickel concentrations. Lead and cadmium were negative influencing factors of blood eGFR in both men and women. Exposure to lead results in amassing proximal renal tubular lining cells in the form of inclusion bodies, which are lead-protein complexes. Cadmium accumulates in renal tubular lining cells bound to a small protein containing 30% cystine. Therefore, the accumulation of lead and cadmium continues to cause kidney toxicity [37].

It is difficult to accurately compare change trends in heavy metals in the body between the data in this study and those reported in previous studies due to differences in the data and raw data. However, a recent study reported that the prevalence of CKD among adults aged 35 years or older living in large cities was 13.8%; CKD showed an increasing trend at an average annual rate of 13.6% over the past five years [38]. Thus, it is important to identify the various causes of decreased eGFR. In view of the fact that diet and lifestyle habits have changed, and the number of patients with chronic diseases such as hypertension and diabetes mellitus is steadily increasing, multilateral efforts should be made to halt the increase in kidney disease. CKD is a disease in which the kidneys are damaged for more than three months, or their function continues to decrease. It is often difficult to detect CKD early because patients do not have any symptoms in the early stages [39]. In this study, the proportion of people diagnosed with hypertension and diabetes was 10.98% and 6.07% respectively if GFR was 90 or higher, but if GFR was less than 60, the proportion of people diagnosed with hypertension and diabetes was 72.15% and 34.18%, respectively, indicating that preventive measures for kidney failure are needed indirectly.

## 5. Conclusions

The study results confirmed the close relationship between heavy metal levels in blood and renal function. This study provides basic data with regard to the correlation and influencing factors between renal function and daily exposure to heavy metals. The study reports a significant association between decreased GFR and heavy metal levels and provides meaningful basic data on the association between heavy metals and renal function. The limitation of this study is the lack of a specific analysis of the various factors affecting eGFR. It is difficult to block all variables because the age, living environment, and health status of the study subjects varied. Therefore, based on this study, a systematic analysis of the effects of heavy metals on renal function by age, environment, and health status is proposed to develop guidelines and educational programs for heavy metals in daily life to promote public health. 

## Figures and Tables

**Table 1:** Baseline characteristics of participants (N = 1984).

Category	Male (N = 920)	Female (N = 1064)	*p*
	N (%), M ± SD	N (%), M ± SD	
Age, year	48.33 ± 16.70	48.20 ± 15.77	0.861
Household income			0.081
Lowest~Lower middle	570 (59.62)	619 (65.23)	
Upper middle~Highest	348 (36.40)	444 (46.79)	
Non-respondent	2 (0.21)	1 (0.11)	
Educational status (years)			0.004
0–9	190 (19.87)	280 (29.50)	
10 or more	676 (70.71)	729 (76.82)	
Non-respondent	54 (5.65)	55 (5.80)	
Smoking	342 (37.2)	54 (5.1)	0.342
Hypertension	202 (22.0)	175 (16.4)	0.002
Diabetes Mellitus	89 (9.31)	80 (8.43)	0.091
Body Mass Index	24.42 ± 3.46	23.51 ± 3.933	<0.001
Blood pressure, mmHg			
Systolic	120.14 ± 14.55	115.07 ± 16.80	<0.001
Diastolic	78.00 ± 10.18	73.35 ± 9.30	<0.001
HbA_1_C, %	5.73 ± 0.911	5.60 ± 0.751	<0.001
Triglyceride	163.76 ± 37.66	109.13 ± 72.21	<0.001
HDL Cholesterol	46.91 ± 11.22	54.99 ± 12.4	<0.001
Lead level, µg/dL	1.85 ± 0.73	1.43 ± 0.60	<0.001
Mercury, µg/dL	4.48 ± 3.37	3.04 ± 2.12	<0.001
Cadmium, µg/dL	0.83 ± 0.49	1.09 ± 0.64	<0.001
Nickel, µg/dL	0.31 ± 0.17	0.34 ± 0.36	0.014
eGFR, mL/min	86.93 ± 17.67	93.07 ± 19.02	<0.001

Abbreviations: HDL, high-density lipoprotein; eGFR, estimated glomerular filtration rate; HbA_1_C, hemoglobin A_1_C.

**Table 2:** General characteristics according to eGFR Stage (N = 1984).

Category	Stage 1 (956)	Stage 2 (949)	Stage 3–5 (79)	*p*
	N (%), M ± SD	N (%), M ± SD	N (%), M ± SD	
Gender				<0.001
Male	388 (40.59)	481 (50.68)	51 (64.56)	
Female	568 (59.41)	468 (49.32)	28 (35.44)	
Age, year	42.06 ± 15.13	52.91 ± 14.78	67.39 ±10.50	<0.001
Household income				<0.001
Lowest~Lower middle	594 (62.13)	565 (59.54)	30 (37.97)	
Upper middle~Highest	361 (37.76)	382 (40.25)	49 (62.03)	
Non-respondent	1 (0.10)	2 (0.21)	-	
Educational status (years)				<0.001
0–9	186 (19.46)	249 (26.24)	35 (44.30)	
10 or more	710 (74.27)	657 (69.23)	38 (48.10)	
Non-respondent	60 (6.28)	43 (4.53)	6 (7.59)	
Smoking	218 (22.80)	165 (17.39)	13 (16.46)	<0.001
Hypertension	105 (10.98)	215 (22.66)	57 (72.15)	<0.001
Diabetes Mellitus	58 (6.07)	84 (8.85)	27 (34.18)	<0.001
Body Mass Index	23.65 ± 3.95	24.15 ± 3.52	24.63 ± 3.62	0.003
Blood pressure, mmHg				
Systolic	114.35 ± 14.77	119.64 ± 16.32	127.70 ± 17.69	<0.001
Diastolic	74.24 ± 9.63	76.97 ± 9.88	73.24 ± 12.84	<0.001
HbA_1_C, %	5.56 ± 0.79	5.72 ± 0.85	6.10 ± 0.95	<0.001
Triglyceride	131.49 ± 129.77	136.02 ± 94.41	151.75 ± 77.06	0.257
HDL cholesterol	51.97 ± 12.36	51.13 ± 12.48	43.62 ± 13.27	<0.001
Lead level, µg/dL	1.48 ± 0.66	1.74 ± 0.66	2.01 ± 0.80	<0.001
Mercury, µg/dL	3.56 ± 2.76	3.89 ± 2.95	3.36 ± 2.90	0.022
Cadmium, µg/dL	0.90 ± 0.58	1.01 ± 0.58	1.27 ± 0.65	<0.001
Nickel, µg/dL	0.32 ± 0.39	0.32 ± 0.14	0.39 ± 0.23	0.096

Abbreviations: HDL, high-density lipoprotein; eGFR, estimated glomerular filtration rate; HbA_1_C, hemoglobin A_1_C.

**Table 3:** Multiple regression analysis according to eGFR.

Variable	B	SE	β	t	*p*	R^2^	F (*p*)
Constant	148.280	6.874	-	21.571	<0.001	0.266	22.160 (<0.001)
Gender	−5.624	1.778	−0.111	−3.162	0.002		
Age	−0.564	0.052	−0.492	−10.888	<0.001		
Educational status	−7.121	1.546	−0.165	−4.606	<0.001		
Household income	−0.216	1.259	0.006	−0.171	0.864		
Current smoking	−3.097	1.255	−0.086	−2.467	0.014		
Hemoglobin A_1_C	0.287	0.613	0.016	0.468	0.640		
Body Mass Index	−0.495	0.171	−0.098	−2.899	0.004		
Systolic blood pressure	0.082	0.053	0.067	1.532	0.126		
Diastolic blood pressure	−0.203	0.075	−0.116	−2.705	0.007		
Lead	−0.764	0.909	−0.030	−0.841	0.401		
Mercury	−0.490	0.186	−0.087	−2.630	0.009		
Cadmium	−1.087	1.130	−0.035	−0.962	0.336		
Nickel	−12.686	3.820	−0.107	−3.321	0.0001		

Dummy variable: gender (female = reference), education status (10 year or more = reference), household income (upper middle~highest = reference).

**Table 4:** Correlation between eGFR and blood lead, mercury, nickel, and cadmium.

Category	Lead	Mercury	Cadmium	Nickel	eGFR
	r (*p*)	r (*p*)	r (*p*)	r (*p*)	r (*p*)
Lead	1				
Mercury	0.262 (<0.001)	1			
Cadmium	0.273 (<0.001)	0.077 (0.001)	1		
Nickel	−0.004 (0.870)	−0.049 (0.028)	0.078 (<0.001)	1	
eGFR	−0.255 (<0.001)	−0.042 (0.061)	−0.164 (<0.001)	−0.056 (0.012)	1

Abbreviation: eGFR, estimated glomerular filtration rate.

**Table 5:** Effects of blood lead, mercury, nickel, and cadmium on eGFR.

		B	SE	β	t	*p*	R^2^	Adj R^2^	F	*p*
Male	Constant	99.166	1.959		50.632	<0.001	0.098	0.095	28.875	<0.001
	Lead	−3.541	0.836	−0.146	−4.237	<0.001				
	Mercury	0.269	0.172	0.051	1.564	0.118				
	Cadmium	−3.329	1.238	−0.092	−2.689	0.007				
	Nickel	−13.437	3.439	−0.126	−3.907	<0.001				
Female	Constant	107.204	1.648		65.050	<0.001	0.058	0.054	14.056	<0.001
	Lead	−6.644	1.019	−0.211	−6.521	<0.001				
	Mercury	0.448	0.270	0.050	1.659	0.097				
	Cadmium	−5.006	0.943	−0.170	−5.306	<0.001				
	Nickel	−1.552	1.546	−0.029	−1.004	0.316				

## Data Availability

The data can be found here: http://knhanes.cdc.go.kr; (Accessed on 4 April 2021).
